# New products

**DOI:** 10.1155/S1463924698000054

**Published:** 1998


Journal of Automatic Chemistry, Vol. 20, No. 1 (January-February 1998) pp. 27-29

### New products

### The Merlin Millennium System

In 1987, P S Analytical launched the first fully automated system for the measurement of mercury at sub ppb levels. Since that time P S Analytical has established a worldwide network of users and a reputation as being the mercury expert. Recently, PSA has launched a successor to the successful Merlin Plus System.

From experience gained in the field and customer feedback, PSA has created the Millennium product range. This range embraces a number of significant improvements to ease the task of the analytical chemist and further advances the Merlin's lead in sensitivity, flexibility and range of sample matrices analysed. The Millennium Merlin System integrates the proven vapour generator and the Merlin atomic fluorescence detector in a simple, compact, ergonomically designed instrument.

The Millennium Merlin is designed for routine use and also offers the range facilities necessary for today's researcher and analytical methods development chemist. It easily meets the current legislation and has the ability to meet the future requirements. The Millennium system is totally controlled from a PC with Windows® based Avalon software compatible the majority of client software, allowing discrete data capture with full integration into user networks and formats. The P S Analytical Millennium Merlin System provides accuracy, precision and has the greatest linear dynamic range of any competitive system, with detection levels of better than 0.1 ppt.

*For further information please contact Paul Stockwell at P S Analytical Ltd, Arthur House, Crayfields Industrial Estate, Main Road, Orpington, Kent BR5 3HP, UK. Tel.: +44 (0) 1689 891211; fax: +44 (0) 1689 896009; email: sales@psanalytical.demon.co.uk*

### The 1999 Keene P. Dimick Award for Chromatography

Nominations are requested for the Keene P. Dimick Award for Chromatography which is presented annually for noteworthy accomplishments in the area of gas and supercritical fluid chromatography (GC, SFC). The award is administered by the Society for Analytical Chemists of Pittsburgh (SACP) and consists of a $5000 cash award presented at a symposium arranged by the awardee during the Pittsburgh Conference week.

Past winners include:

1988 Milton L. Lee—Brigham Young University
1989 Herbert H. Hill—Washington State University
1990 Milos V. Novotny—Indiana University
1991 Harold H. McNair—Virginia Polytechnic Institute and State University
1992 Robert E. Sievers—University of Colorado
1993 Egil J. Jellum—Biochemical Institute, Oslo, Norway
1994 Thomas L. Chester—Proctor & Gamble Corporation
1995 Steven B. Hawthorne—University of North Dakota
1996 Gerhard Schomburg—Max Plank Institute, Ruhr, Germany
1997 Walter Jennings—J&W Scientific

Selection is based on overall accomplishment in the field of GC and/or SFC, including scientific publications, participation in conferences, contribution to science and beneficial influence to other scientists. There are no restrictions of age, nationality, sex or professional affiliation. Deadline for nominations is 30 March 1998.

*Nominations, including a résumé for the candidate, should be sent to: Manny Miller, Chairman, Keene P. Dimick Award Committee, Society for Analytical Chemists of Pittsburgh, 300 Penn Center Boulevard, Suite 332 Pittsburgh, PA 15235-5503, USA.*

### Chromatography data system

The Turbochrom™ Workstation Chromatography Data System provides advanced data handling facilities for gas and liquid chromatography instruments, with all the advantages of Microsoft Windows® 95 and Windows NT®. These include pre-emptive multi-tasking, enhanced software security, and the ease of use of the familiar Windows environment. A unique data protection scheme maintains data collection from all chromatographic instruments, even if the computer, operating system or network connections become inoperative. Of particular interest to pharmaceutical analysts, Turbochrom™ Workstation includes G(A)LP/GMP compliant facilities and a complete suite of validation assistance tools.

*For further information, contact Perkin-Elmer Ltd, Post Office Lane, Beaconsfield, Bucks, HP9 1QA, UK. Tel.: 01494 676161; fax: 01494 679331/3; e-mail: chromg@perkin-elmer.com, Internet http://www.perkin-elmer.com*

### GC/MS

Perkin-Elmer's TurboMass™ is a sophisticated benchtop detector with all the tools needed for both complex and routine GC/MS analyses. Designed as a detector for the AutoSystem XL™ GC, the TurboMass™ will enhance productivity in a wide range of applications, from chemical ionization for pesticide analysis, to detecting organic volatile impurities (OVIs) in drugs by headspace GC/MS. These applications will be of particular interest for pharmaceutical, environmental and petrochemical analyses.

The TurboMass™ offers the highest mass range of any benchtop GC/MS—from 2 to 1200 DA, with a variable scan rate up to 6000 Da/s. Electron Ionization (EI)

0142-0453/98 $12.00 © 1998 Taylor & Francis Ltd produces classical spectra for compound identification, while positive and negative chemical ionization can be added for molecular weight information and more complex samples. The TurboMass™ is available with turbomolecular or diffusion pump, and is compatible with Perkin-Elmer headspace, thermal desorption and large volume injection systems. For ease of use, the instrument runs under the familiar, industry-standard Microsoft Windows NT operating environment.

*For further information, contact Perkin-Elmer Ltd (as above).*

### Support for FT-IR in UK education

Perkin-Elmer developed the first commercial infrared spectrophotometer, the Model 12, in 1944 and has continued to be a major player in spectrometer design, culminating in the current Spectrum series. The company has always had close links with teaching and research laboratories in universities throughout the world. In appreciation, and to celebrate the company's 40th UK Anniversary, Perkin-Elmer has made up to £250 000 available to assist universities and colleges to keep abreast of the latest FT-IR technology. This funding is in the form of a limited number of vouchers, each worth £3000, which can be used towards the purchase of a new FT-IR spectrometer which are being distributed to UK institution. Vouchers and details of FT-IR systems may be obtained from Perkin-Elmer.

*For further information, contact Perkin-Elmer Ltd (as above).*

### Z S P R A Y™

New literature from Micromass (UK) Ltd describes the Z S P R A Y™ interface which is now available on Micromass' Q-Tof, LCT and Quattro-LC product lines. Z S P R A Y™ has been optimized to suit the pharmaceutical industry's requirement for robust, high flow rate electrospray (ES) and atmospheric pressure chemical ionization (APcl) LC-MS interfaces with uncompromised sensitivity. Furthermore, its easily accessible open architecture simplifies NanoFlow ES operation for the analysis of subfemtomole quantities of native polypeptides. A dual orthogonal 'Z' sampling technique [patent pending] delivers the state-of-the-art in contamination avoidance technology, simplicity of operations and enhanced signal to noise.

*For further information contact: Micromass UK Ltd, Queens Avenue, Macclesfield, Cheshire SK10 2BN, UK. Tel.: 01625 434343; fax: 01625 434335; email: http://www.micromass.co.uk.*

### Reacto-Stations™

Stem Corporation has introduced a series of instruments to allow chemists and biotechnologists to easily and

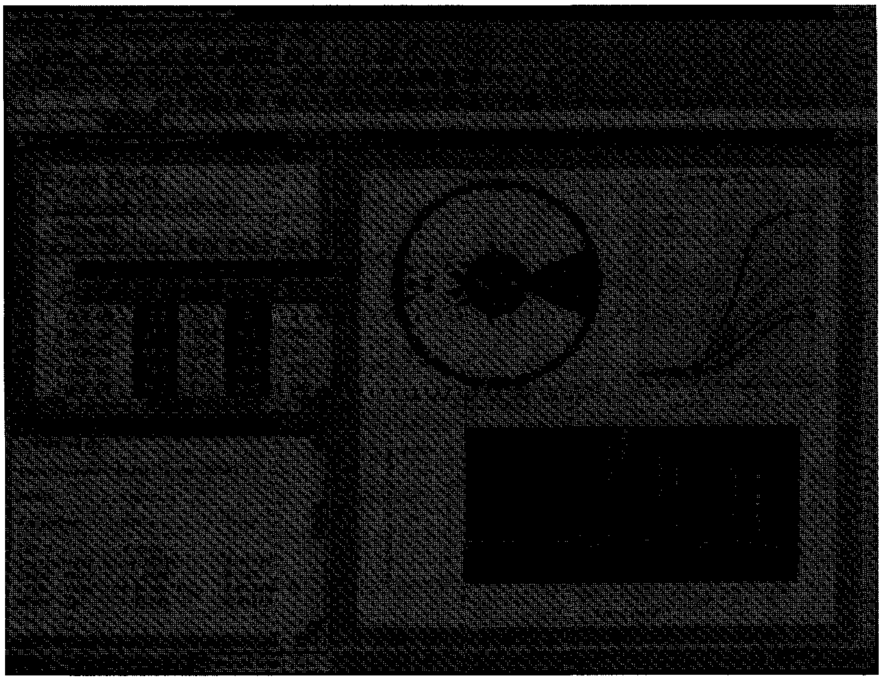

*Origin™ version 5.0, a Windows®-based data analysis and technical graphics software. This release is a substantial improvement of the software including tools for using Microsoft® Excel within Origin, a modern user interface with Microsoft-like tabbed dialogue boxes and dockable toolbars, and extensive use of right mouse-click shortcut menus. Origin 5.0 is an OLE 2 server and offers expanded import and export capabilities. Origin 5.0 is a 32-bit product compatible with Windows 95™ and Windows NT®. Details from Microcal Software, Inc., One Rounhouse Plaza, Northampton, MA 01060, USA. Tel.: 413 586 2013; fax: 413 585 0126; email info@microcal.com.*

inexpensively synthesize compounds of interest. Applications include the fast expanding fields of combinatorial chemistry, organic synthesis, process optimization, enzyme assays, sample concentration and general incubations.

Depending on the model, temperature can be set accurately within the range −30°C–150°C and the sample simultaneously stirred, either gently or right up to 2000 rpm.

Reacto-Stations™ vary in size from 10 to 50 tube versions. The standard tube size is 25 mm diameter, but custom blocks can be produced on request.

Reacto-Stations™ are easy to operate manually for simple experiments or can be integrated within robotic workstations. The whole device can be controlled remotely via RS232/485 interfaces giving the additional facilities of unattended operation, speed variation and temperature ramping.

*For further information contact Ken Browne at STEM Corporation Ltd, Woodrolfe Road, Tollesbury CM9 8SJ, UK. Tel.: 01621 868685; fax: 01621 868445; email: support@stemcorp.com*

### Process automation user conference

**Bernhard Rose**

*Münich, Germany*

Smart manufacturing, optimization of production and how the petro-chemical industry is getting a grip on the costs under pressure of global competition were the main themes at the Aspen World 97 user congress in Boston.

Lawrence Evans, CEO of Aspen Technology, presented the company's 'Plantelligence' to an audience of around 1500 industry managers. Plantelligence is the 'integration of various products' that, following the acquisition of several companies over the last two years, Aspen Technology offers and has put together in a software suite of complementary modules. Plantelligence stands for intelligent solutions for planning, operating and managing process plants in the petro-chemical industry and is comparable with the software offered by SAP for the commercial sector. The focus is on the integration of the individual modules.

Mr Evans said that the petro-chemical industry is facing decisive changes. Globalization is increasing competition at an alarming rate. Excess capacity in the markets is already putting severe pressure on margins. Companies can only improve their profits if they make better use of resources such as feedstock, power and capital.

Just how tense the competitive situation in the oil industry is today was explained by Bernard Bulkin, Director for the International Refinery Business at British Petroleum. In good years, about half of the industry earns money. In bad years, however, this figure falls to about 25%. 'Only those companies that make themselves really fit will survive in the coming years', warned Bulkin. Thus, BP, one of the world's biggest oil companies and refinery operators, anticipates savings of at least 50 cents per barrel of mineral oil through the introduction of modern online control and optimization systems. Given the company's total production of around 1.7 M barrels of mineral oil per day, this would mean savings of more than $300 M every year in the future.

In order to be able to meet the product tolerances and specifications demanded by customers, producers have virtually no choice other than to use sophisticated software for online monitoring, simulation and optimization. Especially in the case of flexebly operated plants with changing production processes, this is decisive for economic production in smaller batches. Through advanced control and online optimization, the German DEA Mineralöl AG has been able to save a million DM per year on blending gasoline. The amortization period: just one year.

The general opinion in the industry is that the use of systems for advanced control and optimization alone would result in savings of 6 to 10% in terms of value-added production. Thus, given an average added value of 40% in the chemical industry, these two processes alone would reduce production costs by some 3 to 4%.

However, this is only a fraction of the actual potential. In the opinion of Lawrence Evans, formerly professor for chemical process technology at MIT, Massachusetts Institute of Technology, in Boston, the 'true potential' for reducing costs with the aid of modern software technology is between 15 and 20% of production costs. 'True potential' refers to the optimization of a chemical process in accordance with its stoichiometric function, modelling this function within the framework of a computer model and operating the plant on the basis of the optimum values calculated. However, even if the target set is only the 'best practice' level, i.e. the elimination of all negative influences in modern plants, it would still be possible to achieve gains of between 10 and 15%. If, for instance, Dupont, a company with revenues of $42 billion, could improve all production processes to 'best practice' standard, this alone would, according to one of the company's managers, lead to savings of around $5 billion per year. If it were raised to the 'true potential' level, the savings would amount to no less than $8 billion, or almost 20% of revenue.

Mr Evans explained that this high potential for improvement is due, among other things, to the fact that it has only been possible in recent years for modern online-optimization software tools to calculate large equation systems with 60 or more variables in real time. The result is that plants can be operated in accordance with an ideal process model (Smart Manufacturing). The process models are developed with the aid of offline modelling tools, which permit processes to be shown interactively on the monitor. In this way, it is possible to display and optimize large-scale plants using mathematical equation systems employing several hundred thousand variables.

*Further information from Aspen Technology, Tel. +32 3 724 0100, e-mail: una.mcnamara@aspentech.com*

